# Mechanical Design and Control System Development of a Rehabilitation Robotic System for Walking With Arm Swing

**DOI:** 10.3389/fresc.2021.720182

**Published:** 2021-11-18

**Authors:** Juan Fang, Kenneth J. Hunt

**Affiliations:** Division of Mechanical Engineering, Department of Engineering and Information Technology, Institute for Rehabilitation and Performance Technology, Bern University of Applied Sciences, Burgdorf, Switzerland

**Keywords:** interlimb neural coupling, arm-leg synchronisation, ground reaction forces, foot loading, impedance control, curved treadmill

## Abstract

**Background:** Interlimb neural coupling implies that arm swing should be included during gait training to improve rehabilitation outcomes. We previously developed several systems for production of walking with arm swing, but the reaction forces on the foot sole during usage of the systems were not satisfactory and there was potential to improve control system performance. This work aimed to design and technically evaluate a novel system for producing walking with synchronised arm and leg movement and with dynamic force loading on the foot soles.

**Methods:** The robotic system included a passive curved treadmill and a trunk frame, upon which the rigs for the upper and lower limbs were mounted. Ten actuators and servocontrollers with EtherCAT communication protocol controlled the bilateral shoulder, elbow, hip, knee and ankle joints. Impedance control algorithms were developed and ran in an industrial PC. Flexible pressure sensors recorded the plantar forces on the foot soles. The criteria of implementation and responsiveness were used to formally evaluate the technical feasibility of the system.

**Results:** Using impedance algorithms, the system produced synchronous walking with arm swing on the curved treadmill, with mean RMS angular tracking error <2° in the 10 joint profiles. The foot trajectories relative to the hip presented similar shapes to those during normal gait, with mean RMS displacement error <1.5 cm. A force pattern that started at the heel and finished at the forefoot was observed during walking using the system, which was similar to the pattern from overground walking.

**Conclusion:** The robotic system produced walking-like kinematics in the 10 joints and in the foot trajectories. Integrated with the curved treadmill, the system also produced walking-like force patterns on the foot soles. The system is considered feasible as far as implementation and responsiveness are concerned. Future work will focus on improvement of the mechanical system for future clinical application.

## Introduction

Arm swing is an integral component of human gait, and should be included during gait training to improve rehabilitation outcomes (1). Lower-limb rehabilitation robotic systems have become increasingly popular, and their efficacy in gait restoration for severely-affected patients have been confirmed by randomised controlled clinical trials (2, 3). However, most systems for walking rehabilitation focus on assisting the legs while the arms are not controlled (4), in spite of the fact that normal gait involves the arms which swing synchronously with the legs. The coordinated arm-leg movement during walking is modulated by interlimb neural circuits. Simultaneously controlled by the central rhythmic networks, arm swing and leg movement in human gait are considered as the most primitive and basic movement patterns (5). Apart from the theory of interlimb neural coupling (6), clinical results also support integrating arm movement into gait rehabilitation. Due to a lack of gait robotic products with arm swing, several other approaches were used for clinical research, such as arm-leg cycling (7), and therapist-assisted arm-swing during locomotion training (8). After 5 weeks of arm cycling training, chronic stroke patients demonstrated stronger interlimb neural coupling, accompanied with enhanced performance of walking and balance (7). Synchronous arm movement during walking on the treadmill facilitated lower limb muscle activation in patients with incomplete spinal cord injury (8). Compared to traditional walking training with the lower limbs only, inclusion of rhythmic arm swing brought more favourable rehabilitation effects in subacute stroke patients (9), with improved balance, sensation, and motor function. Utilising the human interlimb coordination mechanisms is an especially promising area for rehabilitation (10). It is believed that walking with arm swing helps to activate the central rhythmic networks, and thereby enhances neural plasticity (7).

Motivated by the theory and clinical results described above, we previously developed three rotational orthoses for walking with arm swing (11–13), but the reaction forces on the foot sole during walking in those systems were not satisfactory. The initial prototype of a rotational orthosis for walking with arm swing (ROWAS) validated the mechanical feasibility of producing passive arm-leg movement in various positions (11). An automatic ROWAS system was later developed to improve and automate the control system (12). The limitation of these two systems was that air-stepping was produced, where each foot was constantly supported by a shoe platform. Apart from the coordinated movement in the upper and lower limbs, successful walking rehabilitation requires suitable dynamic limb loading to activate the muscular system and the haptic sensory receptors (14). However, it is challenging to produce walking on a fixed ground-simulation plate when the hip, knee and ankle joints are all controlled in passive mode. Therefore, an improved system, ROWAS II, was developed, which implemented an admittance control strategy for active walking, and finally produced “walking” on a ground-simulation plate (13). The participants wore two rigid shoe platforms with wheels at the bottom that rolled on the ground-simulation plate during the stance phase. The exact plantar force pattern on the foot sole was not investigated, but it was believed that the participants in ROWAS II could not obtain dynamic plantar force stimulation at heel strike and toe off as experienced during overground walking. Furthermore, the control system of ROWAS II, which includes three computers for control of eight joints, requires to be improved. It is desirable to develop a new robotic system to produce walking-like dynamic force simulation on the foot soles during walking with arm swing.

A new and improved arm-leg robotic system was developed in this study for future application in neurological rehabilitation and for investigation of interlimb neural coupling. The dynamic loading input from the foot sole is believed to modulate walking patterns and thereby to be beneficial for relearning of walking (15). Furthermore, the elbow joints are also involved while walking normally (16). The new system was therefore required to activate the elbow joints so as to produce proprioceptive sensation feedback in all necessary joints that are involved in normal gait. The requirements of the robotic system include:

- The upper limb (the shoulder and elbow joints) and the lower limb (the hip, knee and ankle joints) are to be actively moved;
- Dynamic loading is to be provided initially on the heel, then the whole foot sole and lastly on the forefoot to mimic ground reaction forces occurring during the stance phase of overground walking;
- A flexible gait pattern is to be produced.

This work aimed to design and technically evaluate a novel system for producing walking with synchronised arm and leg movement and with dynamic force loading on the foot soles.

## Methods

The robotic system was designed using CAD software (SolidWorks, Version 2018, Solid Solutions AG, Zürich, Switzerland) and includes 10 drive units and servocontrollers (maxon motor, Switzerland). The control program was developed using TwinCAT 3 (Beckhoff Automation GmbH & Co. KG, Germany), where the overall system could be operated via a touch panel. Finally, the technical feasibility of the arm-leg robotic system was evaluated using the formal criteria of implementation and responsiveness (17).

### Mechanical Development

**Figure 1:**
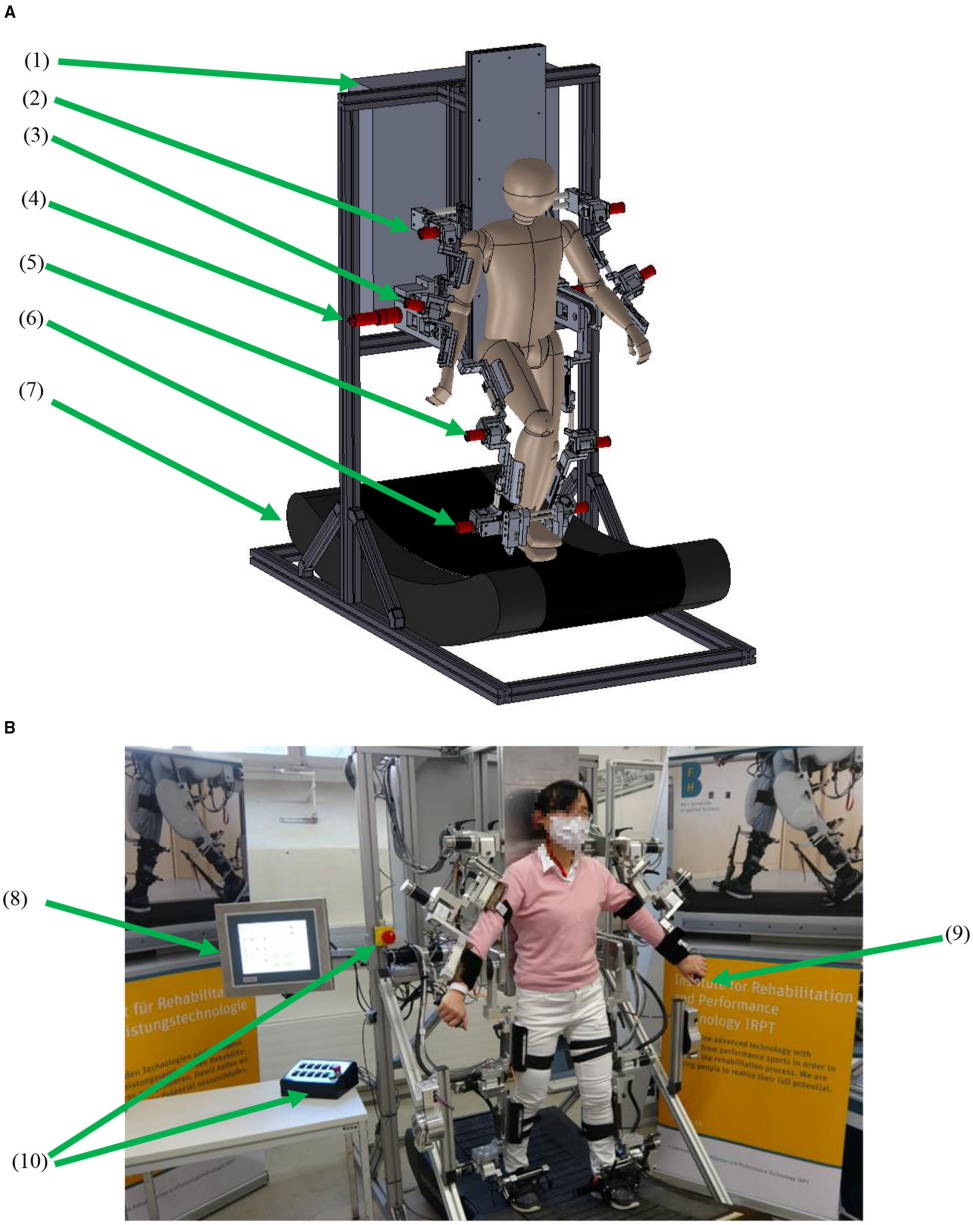
The arm-leg robotic system. **(A)** CAD model and **(B)** prototype. (1) Control unit, (2) shoulder actuator, (3) elbow actuator, (4) hip actuator, (5) knee actuator, (6) ankle actuator, (7) curved treadmill, (8) touch panel, (9) hand-held stop, and (10) stops for operators.

The arm-leg robotic system comprises a trunk frame over a passive curved treadmill and size-adjustable rigs for the upper and lower limbs (Figure 1). The curved treadmill (Woodway GmbH, Germany) provides the user with a stable support during walking. It is an unactuated low-friction curved treadmill (18). The users can stand still on the lowest point of the curved treadmill, which is around in the middle of the treadmill. If the user places the foot on the front part, the treadmill moves due to the curvature. The walking speed is dependent on the step length: if the user walks with a larger step length, then the treadmill speed increases in response.

Selection and mounting of the actuators were based on normal walking performance and the technical requirements of the system. During overground walking, the normalised maximal torques for the shoulder, elbow, hip, knee and ankle joints are ~0.05, 0.03, 0.5, 0.4, and 0.8 Nm/kg, respectively (19–21). However, in the future application of promoting early rehabilitation, the robotic system will be required to provide stepping in a lying position, where a large torque from the hip drive is needed (22). Therefore, two actuator assemblies (DC motor model RE50, gearhead ratio of 236, Encoder HEDL 5540; maxon motor, Switzerland) were used for both hip joints. To provide enough space for arm swing around the hip area, the hip drives were mounted behind the trunk frame (Figure 1), and the power was transmitted via synchronous belts to drive the user's hip joints. Regarding the knee and ankle drives, the user of the system will be encouraged to walk on the treadmill, thereby a proportion of the user's weight would be transferred onto the treadmill during the stance phase. Furthermore, a body-weight support system will be also developed in the future to provide access to neurologically impaired patients. Six actuator assemblies (motor model EC 45, gearhead ratio of 126, MILE encoder; maxon motor, Switzerland) were used for the shoulder, knee and ankle joints. Each elbow joint used the same motor model as used in the shoulder joint, but the gearhead ratio is 66. The drives for the shoulder, elbow, knee and ankle joints were mounted directly aligned with the mechanical joints (Figure 1).

The robotic system was manufactured (Figure 1B). The standing base and the trunk frame are made of aluminium profiles (Bosch Rexroth Corp. Switzerland), while the rigs are made of aluminium alloy. The whole arm-leg robotic prototype is 2.215 m in height and 0.85 m in width. The lowest treadmill surface is 0.405 m above the ground. The arm-leg robotic prototype is designed for users with height ranging between 1.55 and 1.90 m. Each segment length can be manually adjusted so that each joint mechanism can be aligned with users with different anthropometric characteristics. Each joint has mechanical stops to restrict the joint movement within the physiologically safe range (19).

### Overall Control System

**Figure 2:**
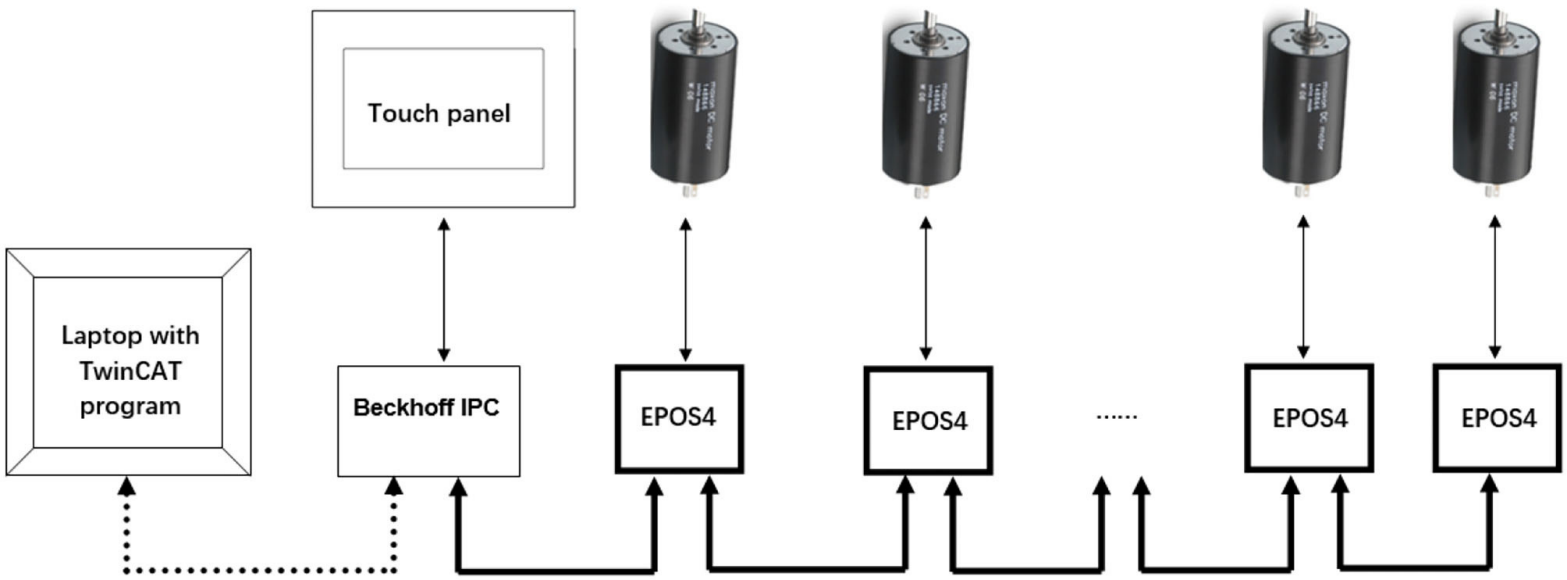
The communication structure. The Laptop downloads the program to the Beckhoff IPC. The system can be operated using the touch panel. The IPC controls the 10 servocontrollers for the upper and lower limbs in real time (Six EPOS4 servocontrollers and their corresponding drives are omitted in the diagram).

Digital positioning controllers with an EtherCAT communication protocol were used for motion control (Figure 2). Two servocontrollers (EPOS4 70/15; maxon motor, Switzerland) controlled two thigh actuators, and eight servocontrollers (EPOS4 50/5; maxon motor, Switzerland) controlled the remaining joints. An industrial PC (IPC, CX5120, Beckhoff Automation GmbH & Co. KG, Germany) runs the control program which calculates the target torque for each motor. Using the Safe Torque Off function in the digital controllers, four emergency stops were implemented (Figure 1B): one emergency stop on a control box on the desk and one emergency stop on the trunk frame, which are for the operator, while another two hand-held stops are for the user to stop the system if he/she feels any discomfort during training. Pressing any of these four emergency stops can immediately set the 10 controllers into the zero-torque-output safe mode.

**Figure 3:**
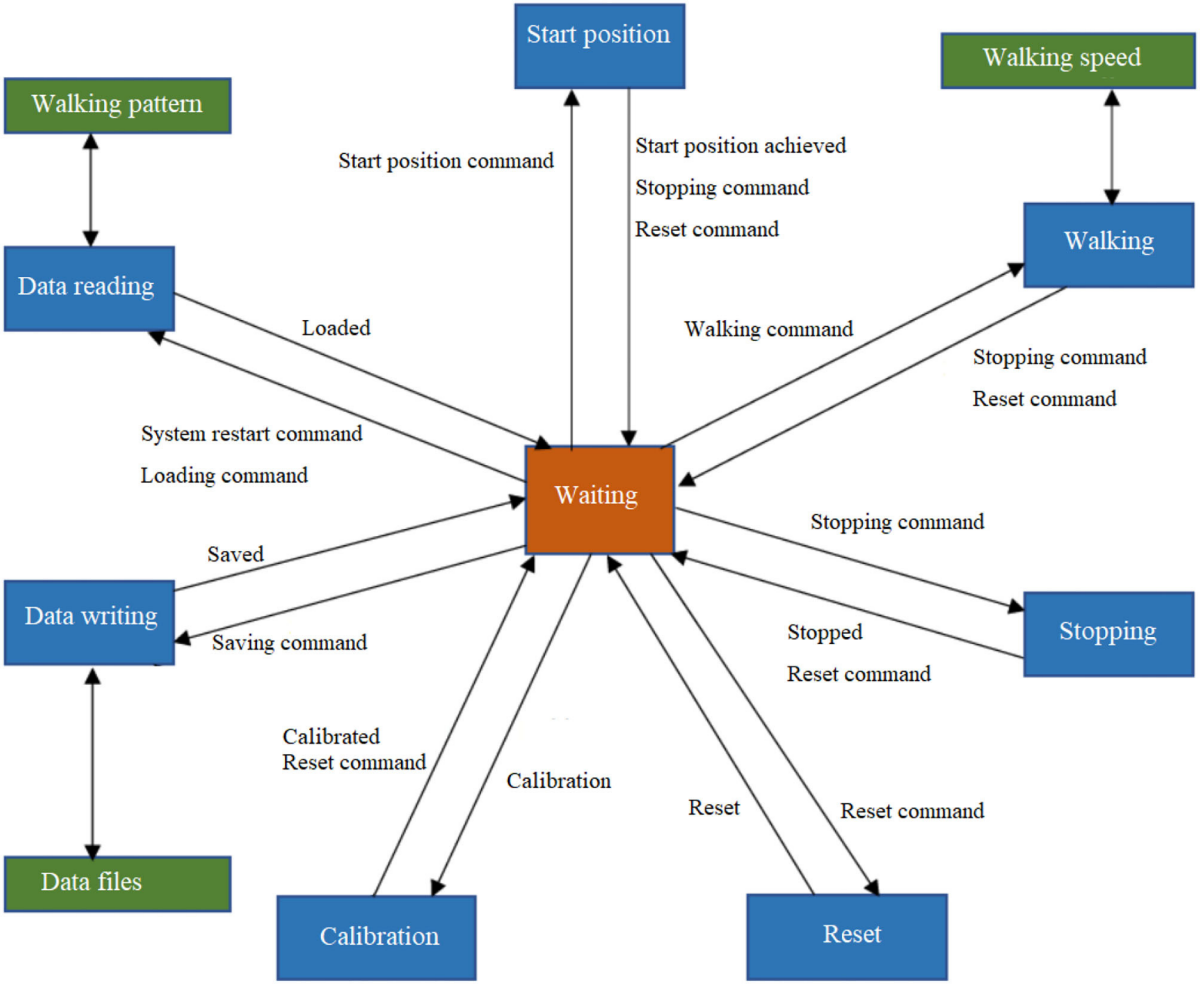
The control functions using state machine blocks.

The control program was developed using state machine blocks in TwinCAT 3 in a laptop, and was downloaded into the IPC. The basic states included (Figure 3): start position, walking, stopping, reset, calibration, reading and saving data. In the waiting state, the system is in an upright standing position. Before starting the walking session, the system calibrates the joint positions, sets the controller parameters, imports the target walking patterns, i.e., the reference joint trajectories. Then, by sending the “start position command,” the system gradually moves the upper and lower limbs to the positions that correspond to heel-strike of the left foot during walking. By sending the “walking command,” the system then produces the walking movement according to the reference gait pattern at any defined speed. When sending a “stopping command” any time during walking, the system gradually reduces the speed and finally stops at the standing-upright position. If any error or problem occurs to the control system, a “reset” command can be sent to clear the errors and set the digital controllers to the waiting state. At the end of training, the controller parameters and the joint motion data during walking are saved by sending the “saving command.”

**Figure 4:**
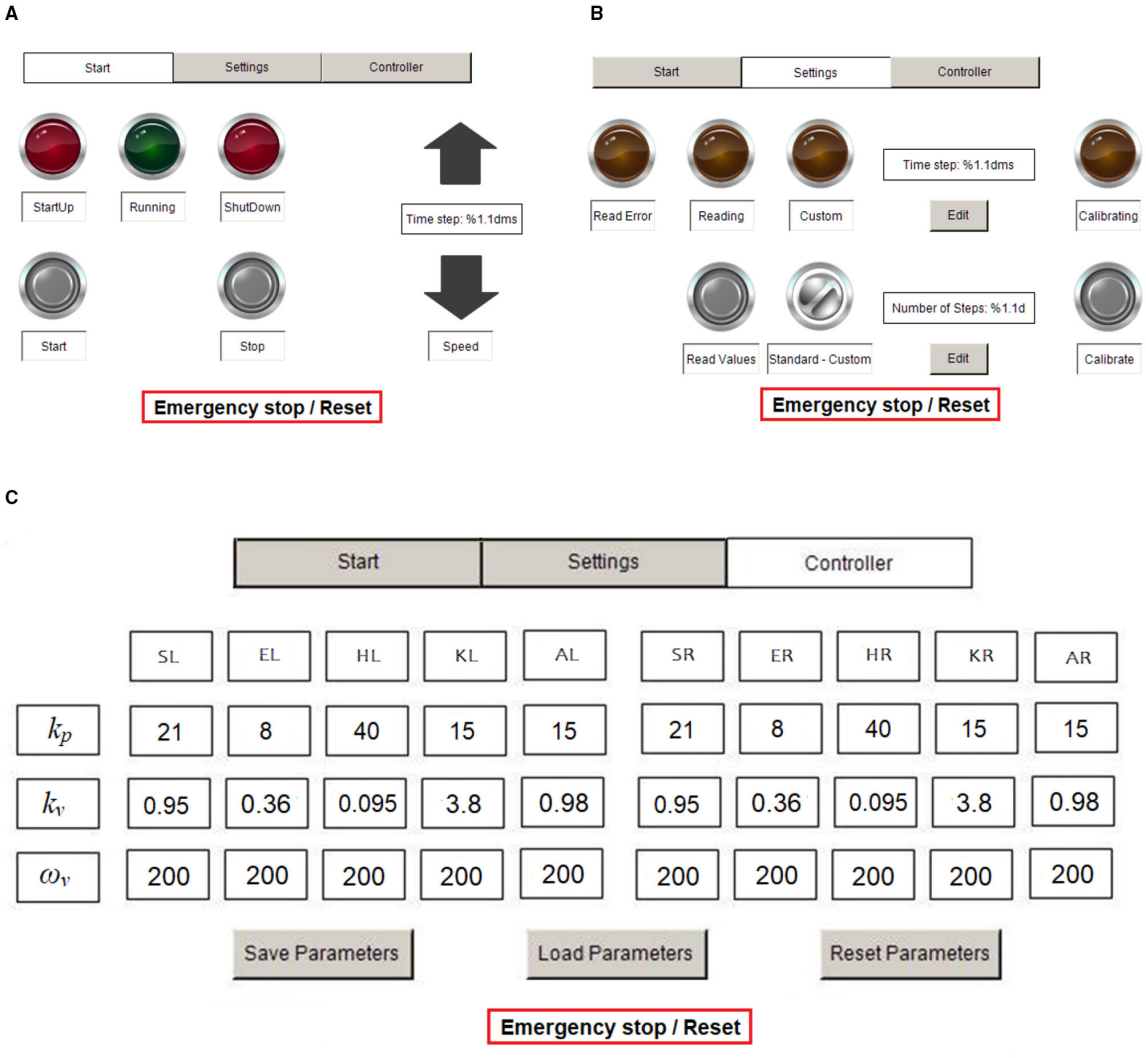
HMI pages for operation. **(A)** Start page, **(B)** settings page, and **(C)** controller page. S, shoulder; E, elbow; H, hip; K, knee; A, ankle; R, right; and L, left.

The touch panel presents three Human Machine Interface (HMI) pages (Figure 4): start, settings and controller pages. Commands such as start and stop of the system and speed adjustment can be sent on the start page. In the settings page, the target movement trajectory of the joints can be imported by pressing “Read Values,” then the home position of each joint can be defined via automatic “Calibration.” The walking speed is adjusted by setting the cadence. The control parameter tuning can be performed in the controller page. The definition of the control parameters is described in section Control Strategy and Outcome Measures.

### Control Strategy and Outcome Measures

**Figure 5:**
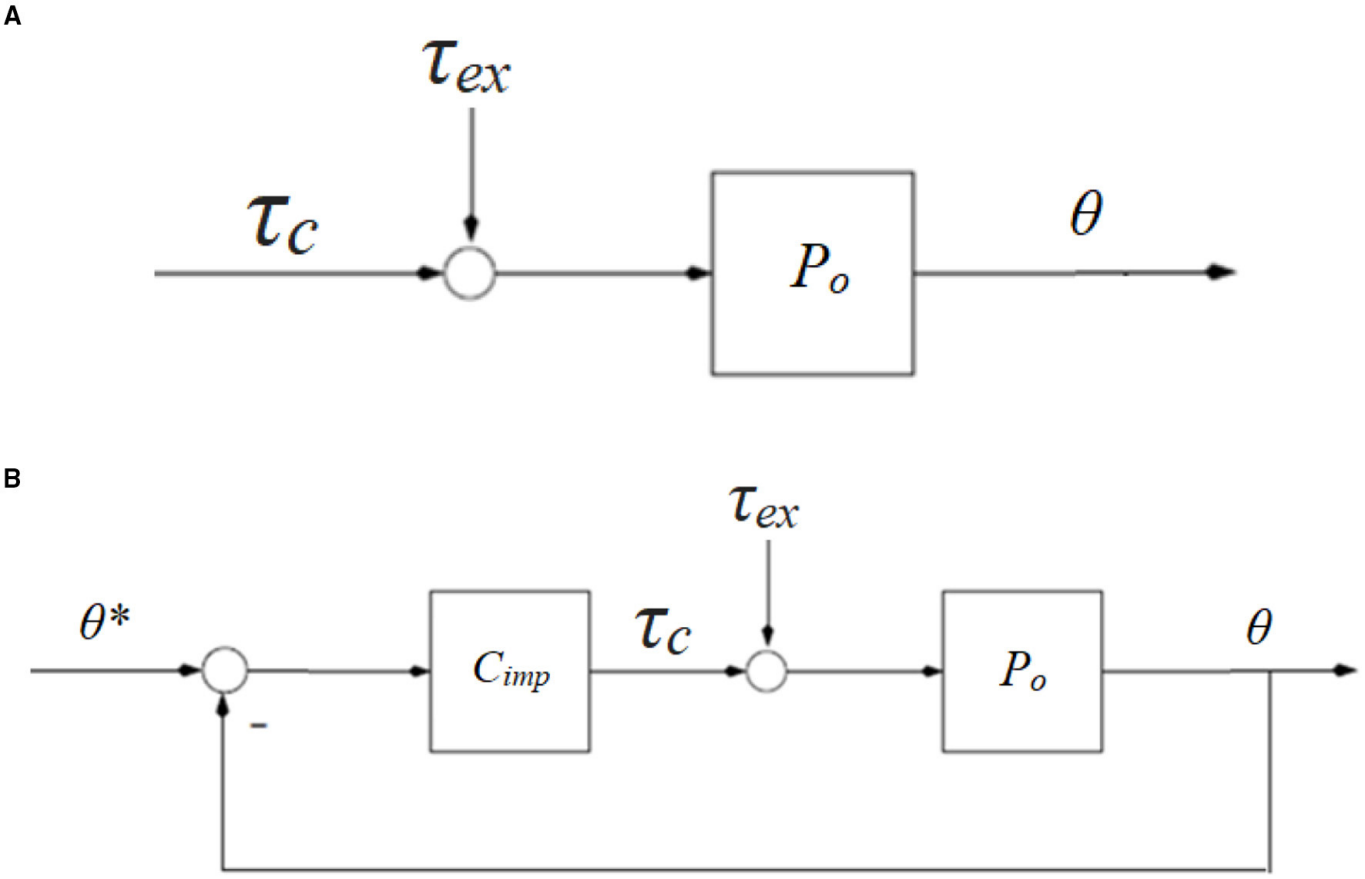
Block diagrams of a human-robotic joint mechnism. **(A)** Open-loop system with external torque τ_*ex*_ and control torque τ_*c*_, and **(B)** closed-loop system with impedance control strategy *C*_*imp*_. θ^*^ is the reference position.

Impedance algorithms were implemented with the servocontrollers running in torque mode. Each joint of the arm-leg robotic system is considered as a spring-damper system with moment of inertia *J*, spring stiffness *k*_*s*_, and damping *k*_*d*_. The user exerts an external torque τ_*ex*_ on the joint during training with the arm-leg robotic system, while the control torque is denoted as τ_*c*_ (Figure 5). The net torque τ_*net*_ is

(1) $$\begin{array}{r} \tau_{net}=\tau_{ex}+\tau_{c}-k_{s}\theta -k_{d}\dot{\theta }. \end{array}$$

Newton's second law yields

(2) $$\begin{array}{r} \ddot{\theta }=\frac{\tau_{net}}{J}. \end{array}$$

Following Laplace transformation,

(3) $$\begin{array}{r} \tau_{ex}\left(s\right)+\tau_{c}\left(s\right)=Js^{2}\theta \left(s\right)+k_{d}s\theta \left(s\right)+k_{s}\theta \left(s\right). \end{array}$$

The transfer function of the spring-damper system, *P*_*o*_, which links the applied torque to the joint position θ, is:

(4) $$\begin{array}{r} \left(\tau_{ex}+\tau_{c}\right)\to \theta :P_{o}\left(s\right)=\frac{\theta \left(s\right)}{\tau_{ex}\left(s\right)+\tau_{c}\left(s\right)}=\frac{1}{Js^{2}+k_{d}s+k_{s}}. \end{array}$$

In the open-loop system, τ_*c*_= 0 (Figure 5A), and the mechanical impedance, *Z*_*ol*_, is

(5) $$\begin{array}{r} Z_{ol}\left(s\right)=\frac{\tau_{ex}\left(s\right)}{s\theta \left(s\right)}=Js+k_{d}+\frac{k_{s}}{s}. \end{array}$$

An impedance controller *C*_*imp*_ (Figure 5B) is used in the closed-loop system as

(6) $$\begin{array}{r} C_{imp}\left(s\right)=k_{v}s+k_{p}, \end{array}$$

where *k*_*v*_ and *k*_*p*_ represent the damping and stiffness of the impedance controller, respectively. Suppose the reference position θ^*^ = 0. Then the output τ_*c*_ from the impedance controller *C*_*imp*_ is:

(7) $$\begin{array}{r} \tau_{c}\left(s\right)=-k_{v}s\theta \left(s\right)-k_{p}\theta \left(s\right). \end{array}$$

Substituting Equation (7) into Equation (3) yields

(8) $$\begin{array}{r} \tau_{ex}\left(s\right)=Js^{2}\theta \left(s\right)+\left(k_{d}+k_{v}\right)s\theta \left(s\right)+\left(k_{s}+k_{p}\right)\theta \left(s\right). \end{array}$$

The transfer function *P*_*cl*_ linking the external torque τ_*ex*_ in this closed-loop system to the joint position θ is therefore:

(9) $$\begin{array}{r} \tau_{ex}\to \theta :P_{cl}\left(s\right)=\frac{\theta \left(s\right)}{\tau_{ex}\left(s\right)}=\frac{1}{Js^{2}+\left(k_{d}+k_{v}\right)s+\left(k_{s}+k_{p}\right)}. \end{array}$$

The mechanical impedance of the closed-loop system is:

(10) $$\begin{array}{r} Z_{cl}=\frac{\tau_{ex}\left(s\right)}{s\theta \left(s\right)}=Js+\left(k_{d}+k_{v}\right)+\frac{k_{s}+k_{p}}{s}. \end{array}$$

Comparison of Equations (4) and (9) yields the changes in the transfer functions after inclusion of the impedance controller. Comparison between Equations (5) and (10) shows that in the closed-loop system, the mechanical impedance is modified by the controller parameters *k*_*p*_ and *k*_*v*_: the closed-loop system has effective stiffness *k*_*s*_ + *k*_*p*_ and effective damping *k*_*d*_ + *k*_*v*_.

The control algorithms were implemented in TwinCAT 3 and ran in the IPC at a sample frequency of 100 Hz. The control parameters *k*_*v*_ and *k*_*p*_ can be adjusted according to the user's impedance requirement. The damping term is implemented in the program with a low-pass filter, where the cut-off frequency ω_*v*_ is selectable, but was set here to 200 rad/s (Figure 4C).

### Evaluation Test and Outcome Measures

Two sets of impedance controller parameters (Table 1) were used with low and high values of gains. The system ran in two situations: (i) without a test person, where the system produced stepping with arm swing with the leg frames stepping in the air; and (ii) with a test person, where the system assisted the person (height of 1.60 m, body mass of 52 kg) to walk on the curved treadmill (see Supplementary Material “Walk in the arm-leg robotic system. mp4”). The movement at arbitrary walking speeds can be produced, but exemplary test results with walking at 71 steps/min are presented in the Results section.

**Table 1:** Impedance parameters.

		**Shoulder**	**Elbow**	**Hip**	**Knee**	**Ankle**
Low	*k_*p*_ (Nm/°)*	21	8	40	15	15
	*k_*v*_ (Nms/°)*	0.95	0.36	3.8	0.98	0.98
High	*k_*p*_ (Nm/°)*	30	10	45	20	20
	*k_*v*_ (Nms/°)*	1.35	0.5	4.5	1.4	1.4

The joint profiles during overground walking at normal cadence from our previous study (23) were used as the target trajectories. To quantify the movement tracking accuracy, root-mean-square error (RMSE) of each joint trajectory was calculated on an evaluation interval from *t*_*o*_ to *t*_1_:

(11) $$\begin{array}{r} \text{RMS}{\text{E}}_{\theta }=\sqrt{\frac{1}{N}\overset{t_{1}}{\underset{i=t_{0}}{\sum }}{\left(\theta_{e}\left(i\right)-\theta_{t}\left(i\right)\right)}^{2}}, \end{array}$$

where *N* is the number of data points in the interval *t*_*o*_ to *t*_1_. θ_*e*_ and θ_*t*_ are the experimental and target joint angles, where the shoulder, elbow, hip, knee and ankle joints are considered.

Using the hip segment position as origin (0, 0) of the reference frame, the segmental trajectories of the ankle (*X*_*ankle*_, *Y*_*ankle*_) and toe (*X*_*toe*_, *Y*_*toe*_) were calculated using:

(12) $$\begin{array}{r} X_{ankle}=l_{t}\sin\theta_{h}+l_{s}\sin\left(\theta_{h}-\theta_{k}\right), \end{array}$$

(13) $$\begin{array}{r} Y_{ankle}=-l_{t}\cos\theta_{h}-l_{s}\cos\left(\theta_{h}-\theta_{k}\right), \end{array}$$

(14) $$\begin{array}{r} X_{toe}=X_{ankle}+l_{f}\sin\left(90-\theta_{h}+\theta_{k}-\theta_{a}\right), \end{array}$$

(15) $$\begin{array}{r} Y_{toe}=Y_{ankle}+l_{f}\cos\left(90-\theta_{h}+\theta_{k}-\theta_{a}\right). \end{array}$$

where *l*_*t*_ and *l*_*s*_ are the lengths of the thigh and shank segments, while θ_*h*_, θ_*k*_, and θ_*a*_ are the angles for the hip, knee and ankle joints. The difference between the target and actual segmental trajectories was analysed by calculating RMSE when the target and actual segment trajectories were used in Equation (11).

To monitor the plantar pressure distribution during walking using the robotic system, a pair of wireless flexible foot insoles (stAPPtronics, Vorarlberg, Austria) was used, which records the pressure under 12 different areas of each foot sole. The pressure data were analysed and saved using the research software package (stappone's app, stAPPtronics).

To assess the technical feasibility of the arm-leg robotic system, the formal criteria from (17) were used: (i) implementation—could the system be easily operated to produce the walking-like movement? and (ii) responsiveness—was the measurable movement close to the target, as far as the joint and foot trajectories were concerned? The arm-leg robotic system was considered to be responsive if the tracking errors in joint and segmental trajectories were <5° and 5 cm, respectively.

## Results

**Figure 6:**
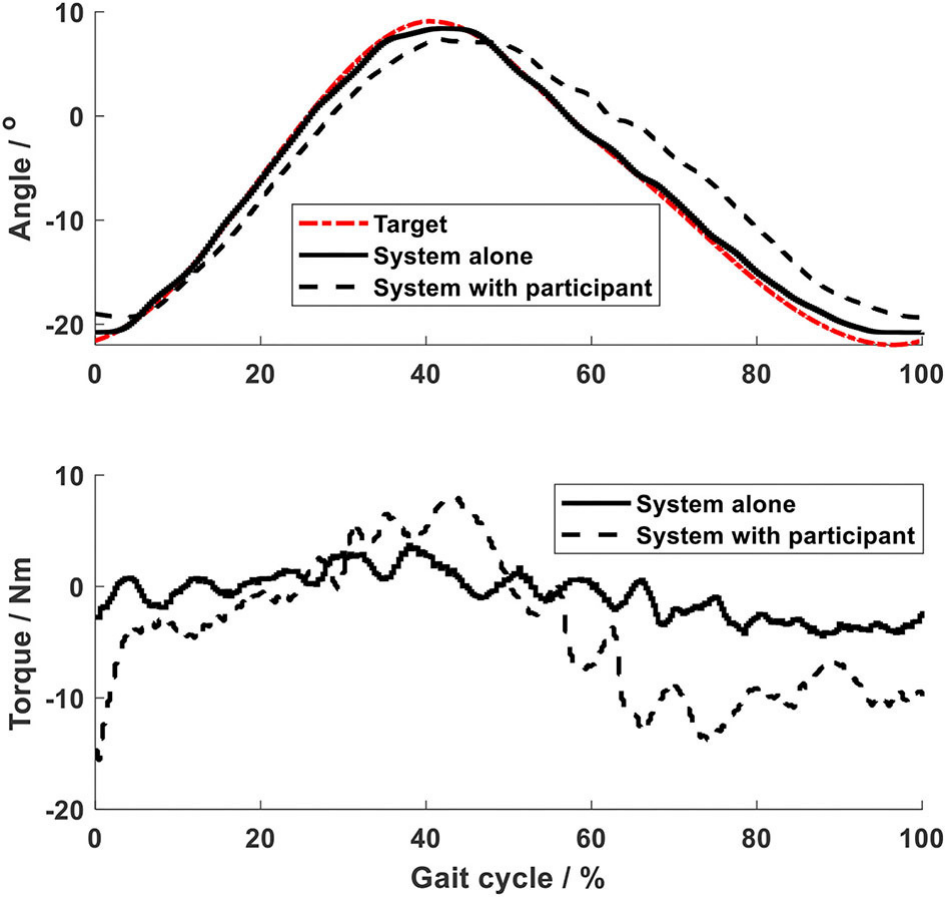
Shoulder joint.

**Figure 7:**
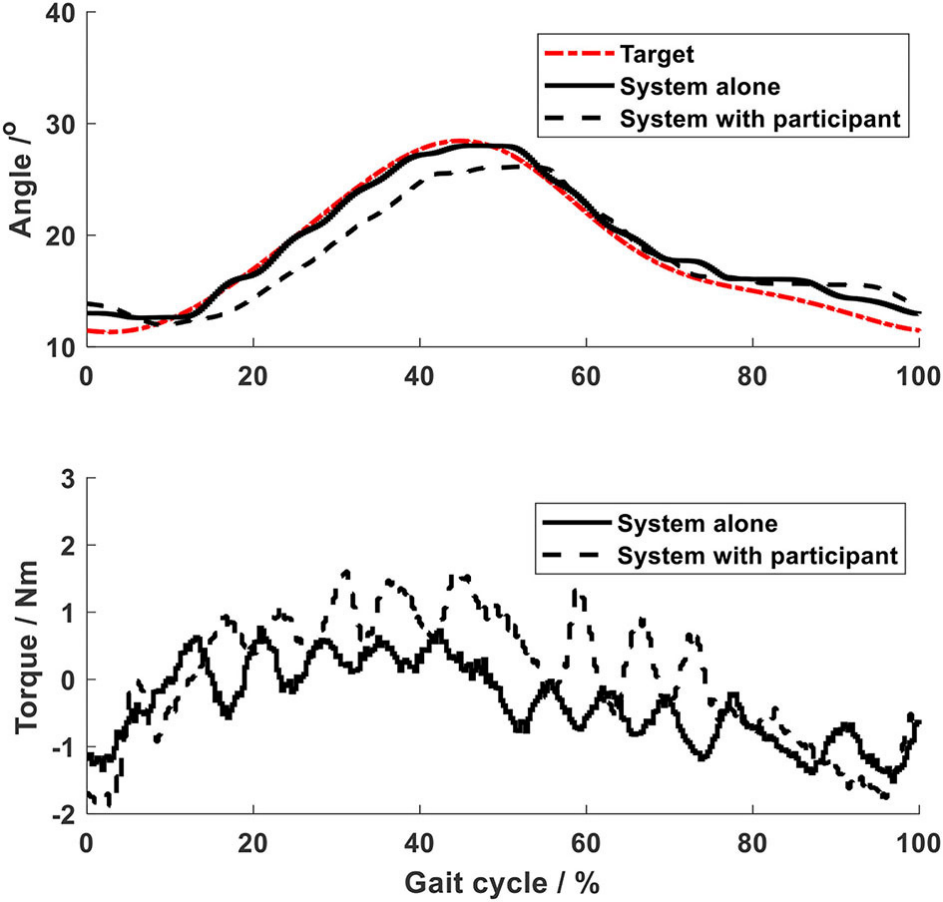
Elbow joint.

**Figure 8:**
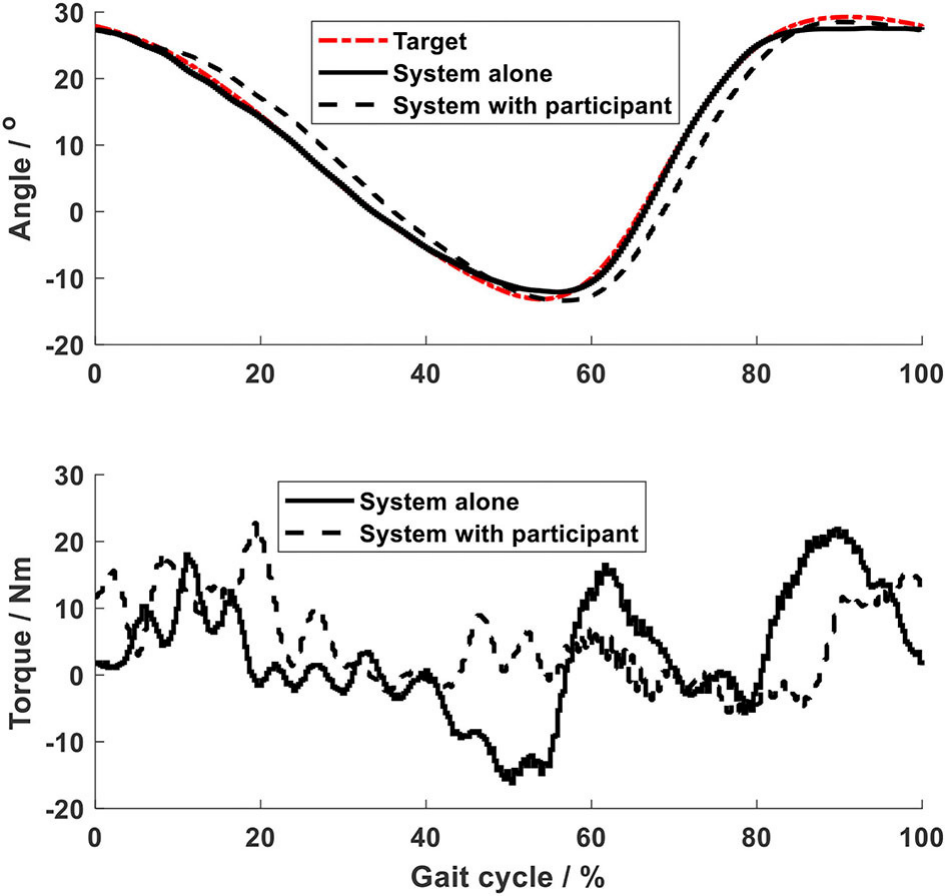
Hip joint.

**Figure 9:**
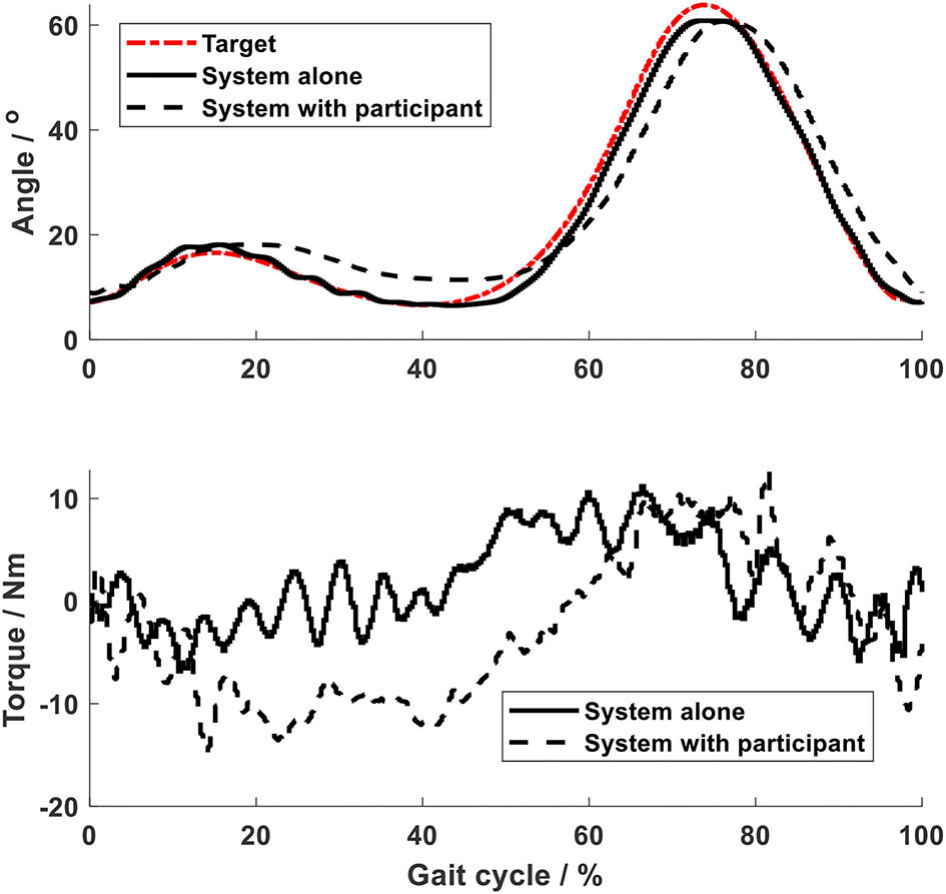
Knee joint.

**Figure 10:**
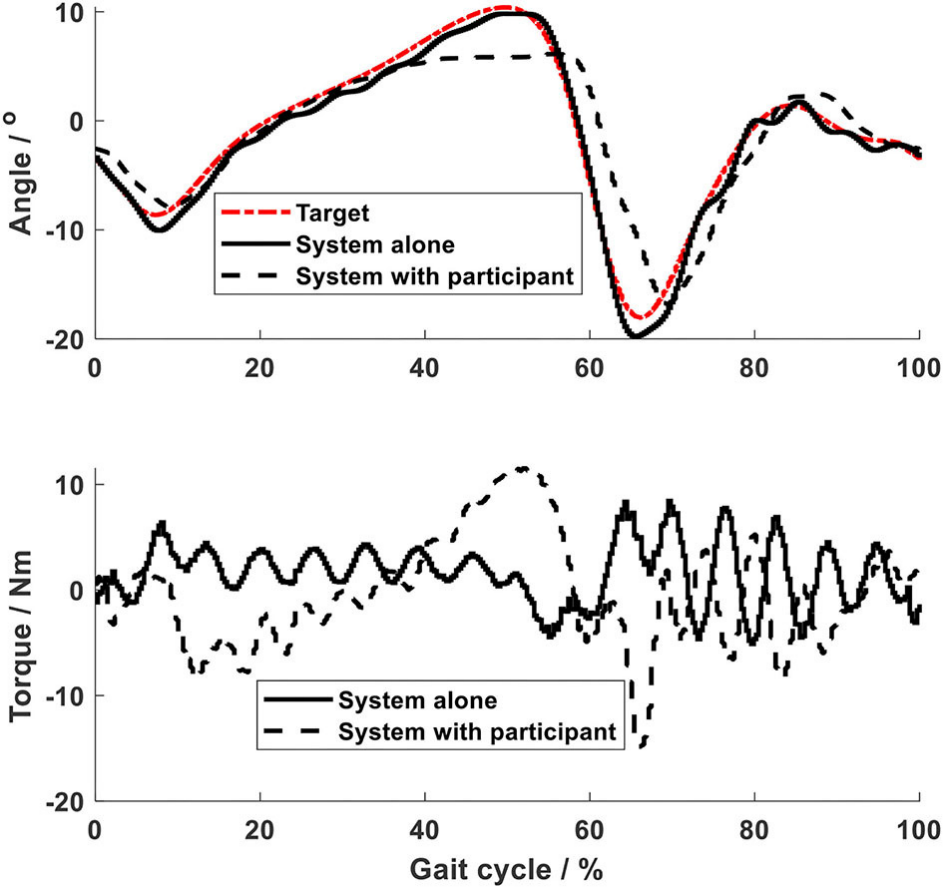
Ankle joint.

Using the impedance algorithms with low values of gains (Table 1), the arm-leg robotic system alone produced walking-like movement (solid lines in Figures 6–10). The joint trajectories were similar to the reference (red dash-dot lines), with the RMSE for the shoulder, elbow, hip and ankle joints smaller than 1° and for the knee joint smaller than 2° (Table 2). The high tracking accuracy (overall mean RMSE of 1°) of the 10 joint trajectories showed that the robotic system with the low values of impedance controller parameters produced synchronous arm-leg walking.

**Table 2:** RMSE of the joint and segmental trajectories.

		**System only (low-gain parameters)**	**System with test person (low-gain parameters)**	**System with test person (high-gain parameters)**
Joint trajectories (°)	Shoulder	0.64	2.13	2.05
	Elbow	0.98	1.54	1.28
	Hip	0.80	0.62	0.49
	Knee	1.76	2.80	2.13
	Ankle	0.97	1.72	1.94
	Mean ± SD	1.03 ± 0.43	1.76 ± 0.80	1.58 ± 0.69
Foot trajectories (m)	Ankle_x	NA	0.020	0.014
	Ankle_y	NA	0.008	0.001
	Toe_x	NA	0.022	0.017
	Toe_y	NA	0.008	0.008
	Mean ± SD	NA	0.015 ± 0.008	0.010 ± 0.007

*NA, not applicable*.

**Figure 11:**
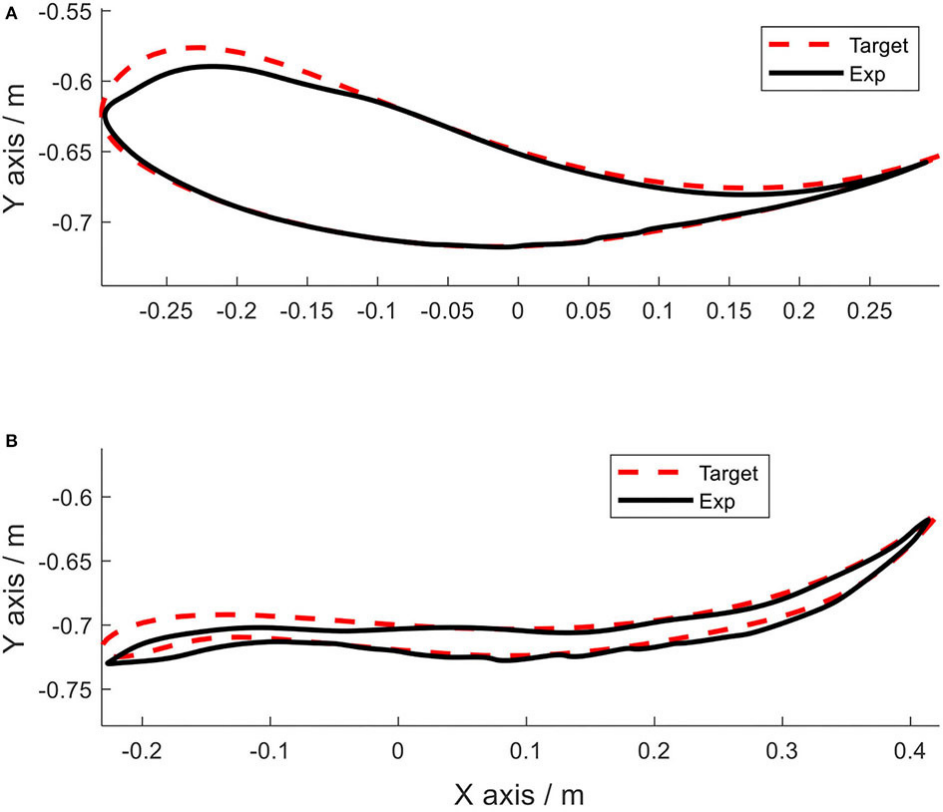
Foot trajectories. **(A)** Ankle and **(B)** toe.

**Figure 12:**
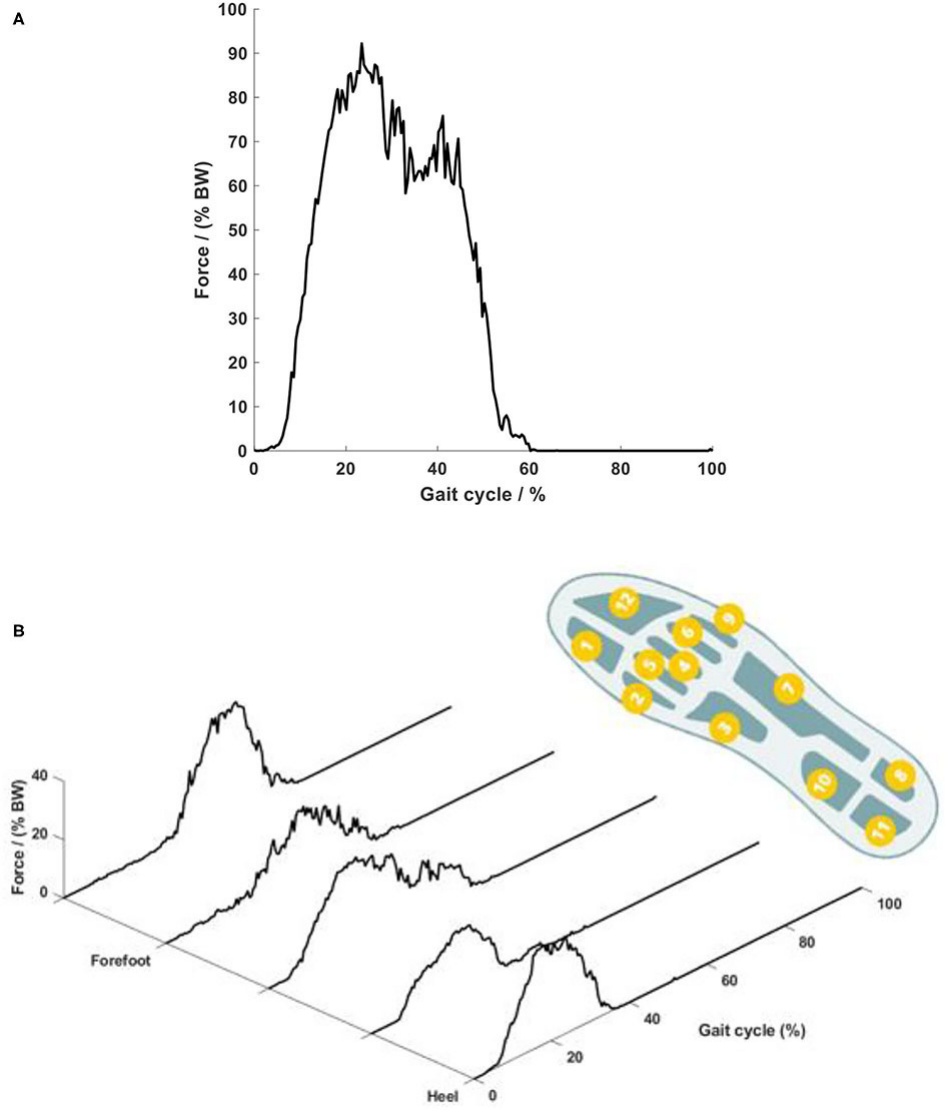
Plantar force on the foot sole. **(A)** Temporal curve of the overall force and **(B)** force on different plantar areas. The right foot sole shape shows the location of the 12 pressure sensors. Each curve in the plot shows the summation of the pressures in the four corresponding foot areas.

Using the same impedance controllers with low-gain parameters, the system assisted the test person to walk on the curved treadmill (Figures 6–12). Although the test person walked actively, the torque required for each joint increased (dashed lines in Figures 6–10). The shoulder, hip and knee joints presented similar range of motion to the target, while the elbow and ankle joints showed 2.4° and 4.4° smaller peak values during 50% of the gait cycle (GC). The elbow deviation was believed to come from the voluntary input of the test person, while the reduced foot dorsiflexion was considered to occur due to the mechanical constraints from the curved treadmill. Compared to the system alone (i.e., without test person), the inclusion and active participation of the test person resulted in large RMSE values in all joints, with a mean RMSE of 1.76° (Table 2). In spite of the joint deviations, the ankle and toe trajectories relative to the hip (Figure 11) presented similar trajectories to normal gait. The toe trajectory in the early-stance phase is more curved compared to the late-stance phase (Figure 11B), which follows the standard toe trajectory in normal gait (23). The toe and ankle trajectories were generally close to the target, with mean difference <1.5 cm (Table 2).

The force on the foot sole during walking with the robotic system presented typical double-bump characteristics, which is similar to that occurring in overground walking (21). The force during a typical walking session with the low-gain parameters is presented in Figure 12. The force started at heel strike, achieved the first peak of 92% body weight (BW) during the early stance phase (20% GC), then reduced to 61% BW during the mid-stance phase (20 to 40% GC), and lastly showed the second peak of 75% BW during the late-stance phase (40% GC) (Figure 12A). The second peak showed a lower amplitude than the first peak, which might be because the test person exerted less propulsion force during the late stance phase due to the robotic assistance. It should be noted that during slow overground walking, the peaks of the ground reaction forces, including the second peak, are often observed to reduce (24). Close observation of the scattering pattern of the plantar force (Figure 12B) shows that during the early stance phase (0 to 20% GC), the force occurred on the heel area only; and then at the mid-stance (20 to 40% GC), the force was scattered over the whole plantar area, including the heel, middle foot and forefoot. During 40 to 60% GC, the force around the heel area reduced to zero, while appearing mainly on the forefoot area. The peak force on the top forefoot area at the end of the stance phase increased up to 40% BW. This plantar force pattern agrees with the sequence of weight-bearing pressures in the literature (16).

Increasing the impedance parameters made the robotic system stiffer and resulted in substantially reduced RMSE for all trajectories (Table 2): using the high-gain impedance controllers, mean RMSE of the 10 joint trajectories reduced from 1.76 to 1.58° and mean RMSE of the foot trajectories reduced from 1.5 to 1.0 cm (Table 2).

The test person reported that they felt as if they were walking. In the first session with the low-gain impedance parameters, she needed some time to know how she should move so as to synchronise with the system. After she became used to the system, she felt obvious stance and swing phases, judging from the arm-leg movement and especially the load on the foot. In the high-gain impedance walking session, she recognised the walking phases immediately. The test person reported the overall test comfortable, although she felt a somewhat constrained arm movement during the whole test.

## Discussion

The aim of this work was to design and technically evaluate a novel system for producing walking with synchronised arm and leg movement and with dynamic force loading on the foot soles. The principal design requirements of the system were synchronised arm-leg movement, dynamic foot loading and flexible gait patterns. Using automatic control of 10 joints, the system produced synchronised arm-leg movement. Impedance control algorithms enabled the system to assist the test person to walk on the curved treadmill and dynamic foot loading was produced. The synchronous arm-leg movement and the dynamic forces on the foot soles were similar to those occurring during overground walking. This arm-leg robotic system was designed to serve as a device for investigation of interlimb neural coupling, and also as a testbed for neurological rehabilitation of walking.

The robotic system showed advantages of easy operation and convenient control development. The digital positioning controllers used the EtherCAT communication protocol, which allowed real-time multi-axis motion control via an IPC. The HMI on the touch panel provided for convenient start and stop of the system, and also allowed easy tuning of the control parameters. The operator-friendly HMI included comparable functions to what the user interfaces in a typical rehabilitation robotic system provide (25), and give substantial advantages compared to our previous systems (11–13).

The movement of the arm-leg frames from the system alone, without a test person, was similar to the reference trajectories with mean RMSE in 10 joints of 1°, which demonstrated that the first design requirement was met. The flexible topology of the control system makes it convenient for future expansion, for example using additional drives for body-weight support system. The synchronous arm-leg walking patterns were implemented, which met the technical feasibility criterion of implementation.

The arm-leg robotic system enabled walking on the integrated curved treadmill, and produced dynamic loading on the foot soles. The curved treadmill provided a stable support to the user during the stance phase. By transferring the body weight to the treadmill, the required power of the drive units during the stance phase, especially of the ankle units, was reduced. Furthermore, the support from the treadmill during the stance phase produced dynamic forces on the foot sole. The plantar force pattern on the foot sole is of a similar double-bump pattern to overground normal walking, with the force starting at the heel and finishing on the forefoot. The second design requirement was met. It is of great interest to compare the actual force on the foot sole during walking on the curved treadmill with the ground reaction forces during overground walking: this will be further investigated in the future.

Impedance control algorithms enabled the system to assist walking on the curved treadmill, which provided the basis of the generation of a curved toe trajectory. Normal overground gait patterns includes a rhythmical oscillation of the body trunk/hip joint centre as well as synchronised movement in the arm and legs. In order to assist walking training overground (26) or on the treadmill (27), gait robotic systems often develop a dynamic mechanism to assist vertical rhythmic oscillation of the trunk. Such a dynamic trunk support mechanism was not developed in this study in order to simplify the orthosis structure. Instead, oscillation of the body trunk/hip joint centre during normal walking was compensated by using the curved treadmill. Integration of the curved treadmill was based on our observation of the curved toe trajectory relative to the hip (23). It was believed that if the test person walked on a curved treadmill, then the trunk movement would be vertically stabilised. This concept was validated by visual observation of the trunk movement (see Supplementary Material “Walk in the arm-leg robotic system. mp4”). As the curved toe trajectories are slightly different between individuals (23), and might not necessarily be of the same shape as the curved treadmill, this study controlled the joint trajectories of the arm and legs using impedance algorithms, which met the third design requirement. Furthermore, the impedance controllers allowed active participation of the user during the training. Depending on the control parameters, the system had different impedances, allowing different deviations of the joint trajectory from the target. The results showed that with high-gain control parameters, the actual trajectories are substantially closer to the target. It is interesting to analyse the active participation from the user, which will be further investigated after inclusion of force sensors. The system was considered responsive, as far as the walking patterns with different impedances is concerned.

This arm-leg robotic system will be a useful tool to investigate interlimb neural coupling. Intensive research on neural interaction during the coordinated arm-leg movement has been based on the daily activities of sitting (28), standing (29), level walking (30), inclined walking and stair climbing (31). In order to investigate the properties of interlimb neural coupling, and especially in those patients who cannot perform such daily activities independently, several assistive devices were used, such as the arm-leg cycle ergometer (7), a reciprocal arm-leg apparatus (32), and a recumbent stepping machine that mechanically coupled the handles and pedals (33). Despite generation of synchronous movement in the arms and legs, these devices produced movement which was different from normal walking. The system developed in the current study produced an arm-leg movement pattern which was kinematically and kinetically similar to overground walking. The system serves as a good testbed for further investigation of interlimb neural coupling in people with different neurological impairments. Research topics such as the influence of passive/active arm movement in the muscle activity of the stationary/passive/active leg in the ipsilateral/contralateral side of the body can be extensively investigated. The control algorithms accompanied with the HMI enable the system to accommodate for people with varying degrees of muscle weakness and to promote active use of the limbs whenever possible. For example, for hemiplegic stroke patients, the control parameters for the left and right sides can be specifically tuned so as to provide more assistance for the more-impaired side, and less assistance for the less-affected side. The controller HMI page allows easy parameter tuning, which enables the system to be conveniently set up for patients with individual assistance requirements.

The developed system will be a novel system for walking rehabilitation with arm swing, and simultaneously a testbed to search for training parameters that optimise the rehabilitation process. During walking rehabilitation with arm swing, the walking speed and the arm movement pattern are important training parameters. It was observed that, during recumbent stepping, faster upper limb movement facilitated neuromuscular recruitment of lower limb muscles (33). During arm-leg cycling, it was recommended that a minimum arm cycling frequency of about 0.8 Hz be used for activation of the leg muscles (34). However, gait training is often implemented at a slow speed clinically, due to patients' physical restrictions. Normal gait analysis revealed that during walking at comfortable and fast speeds, arm movement was in phase with the contralateral hip, whereas during walking at speeds lower than a certain range, both upper limbs swung forward and back in unison at twice the stride frequency of the lower limbs (35–37). The varying arm-leg movement pattern at different speeds might imply a speed-modulated interlimb neural interaction. A study on arm-leg cycling in able-bodied participants (38) observed that apart from the cycling speed, the arm movement frequency also influenced the muscle activity in the legs. Such results, in spite of yielding hints to the setting of suitable training parameters, could not be directly applied to patients, because neurological impairments might alter the interlimb neural coupling in patients. It was observed that patients with Parkinson's disease showed reduced shoulder-hip movement coordination during walking when compared to abled-bodied participants (39). Patients after incomplete spinal cord injury could not produce an arm-leg frequency ratio of 2:1 during walking at slow speeds (40). Based on those observations, the training parameters such as walking speed and arm synchronisation frequency need to be further investigated in patients for the best rehabilitation effect. The developed arm-leg system allows automatic setting of the speed via the HMI page. Furthermore, the new target movement pattern can be imported to the system via the setting page, if a different arm-leg movement frequency is required. This operator-friendly HMI allows to provide a speed-modulated arm-leg movement pattern for future investigation of suitable training parameters.

The strengths of this study include: (i) mechanical integration of the curved treadmill into the robotic system, which provides the basis for the generation of the curved toe trajectory and also dynamic foot loading; (ii) impedance control algorithms, which enables production of arm-leg walking based on automation control technology; and (iii) easy operation interface, which enables convenient control parameter tuning and training movement setting. The system produced coordinated arm swing during walking, with kinematics and kinetics similar to normal gait patterns. The results from one test person are adequate to evaluate the technical feasibility in implementation and responsiveness, but are insufficient to show the feasibility in populations with different neurological deficits. This limitation will be addressed by investigating the user acceptability of this system after the future work described below is completed. A further limitation was that visual feedback was not provided to the participant. Without information on the target and the actual walking pattern, it was reasonable that the test person required a certain time to get used to the system, especially during the session with the low-gain impedance parameters, where the assistance from the system was limited. Due to the dimensions of the hip drive mechanism, arm swing was achieved with a shoulder abduction of about 25°. That was why the test person reported “a somewhat constrained arm movement” during the whole test. This limitation could be addressed by using a smaller hip drive.

This work focused on the mechanical design and control system development based on automation control technology. The next step is to evaluate the acceptability of the robotic system with different users. Further mechanical work will be performed so that the system can provide access to people with neurological disorders. The trunk frame should be able to be tilted down for production of stepping in a supine position. A body weight support system is desirable for potential clinical application. Force sensors are to be included in the device to measure the voluntary inputs of the upper and lower limbs. This system serves as a device for future investigation of interlimb neural coupling, and also as a testbed for neurological rehabilitation of walking. Apart from neurological recovery, cardiopulmonary fitness is also an issue where rehabilitation robotic systems have great potential (41). The developed arm-leg robotic systems will be applied in cardiopulmonary testing and training in the future.

## Conclusions

A novel arm-leg robotic system was designed and constructed. State-of-the-art automation technology enabled the system to produce walking on the curved treadmill with synchronous leg movement and arm swing including shoulder and elbow activation: the robotic system produced walking-like kinematics in the 10 joints and in the foot trajectories. Integrated with the curved treadmill, the system produced plantar stimulation and force patterns on the foot soles which were similar to normal overground gait. The system is considered feasible as far as implementation and responsiveness are concerned. Future work will focus on improvement of the mechanical system for future clinical application.

## Data Availability Statement

The raw data supporting the conclusions of this article will be made available by the authors, without undue reservation.

## Ethics Statement

Ethical review and approval was not required for the study on human participants in accordance with the local legislation and institutional requirements. The patients/participants provided their written informed consent to participate in this study.

## Author Contributions

JF and KH developed the system concept and the control algorithms. JF conducted the experiments, analysed the data, and drafted the manuscript. KH revised it critically for important intellectual content. Both authors read and approved the final manuscript.

## Conflict of Interest

The authors declare that the research was conducted in the absence of any commercial or financial relationships that could be construed as a potential conflict of interest.

## Publisher's Note

All claims expressed in this article are solely those of the authors and do not necessarily represent those of their affiliated organizations, or those of the publisher, the editors and the reviewers. Any product that may be evaluated in this article, or claim that may be made by its manufacturer, is not guaranteed or endorsed by the publisher.
